# Long-term remission after multiple bone metastases following cervical cancer: A case report^☆^

**DOI:** 10.1016/j.gynor.2013.03.003

**Published:** 2013-03-18

**Authors:** Hiroshi Tsubamoto, Kayo Inoue, Yuji Ukita, Yoshihiro Ito, Riichiro Kanazawa

**Affiliations:** Department of Obstetrics and Gynecology, Hyogo College of Medicine, Japan

**Keywords:** Bone metastases, Cervical cancer, Bisphosphonate, Chemotherapy

## Abstract

• Multiple bone metastases following cervical cancer was completed resolved.
• Metastatic lesions were present within a previously irradiated zone of primary external radiation.
• Concurrent chemotherapy and bisphosphonate administration is promising.

## Introduction

Cervical cancer is a leading cause of cancer-related death worldwide. The most common sites for the hematogenous spread of cervical cancer are the lungs, liver, and bone (Hong et al., 2004; Hage et al., 2000). Clinical incidence of bone metastases was reported in 3.8% of 1053 patients with cervical cancer by Nobunaga et al. (1988) and in 4.4% of 1252 patients with cervical cancer by Blythe et al. (1975). Symptomatic bone metastases cause severe pain or pathological fractures, which drastically decrease the patient's quality of life. In an autopsy study, Abdul-Karim et al. (1990) and Disibio and French (2008) reported the incidence of bone metastases from cervical cancer to be 6.3% and 8.6%, respectively. Moriwaki noted bone metastases in 18.6% of 6109 cadavers with primary cervical cancer from a report of the Japanese Society of Pathology (Moriwaki, 2007).

Early detection and proper palliative care, such as treatment with bisphosphonates (BPs) or denosumab, could help avoid these skeletal-related events. In cervical cancer, treatment of bone metastases is usually aimed at palliation and not prolongation of patient survival (Thanapprapasr et al., 2010). Numerous clinical and preclinical studies have examined the anti-cancer effect of BPs (Gnant and Clézardin, 2012); however, to the best of our knowledge, there are no reports regarding their use in cervical cancer. Here, we present a case of multiple bone metastases arising after concurrent chemoradiation for cervical cancer in a patient with long-term disease-free survival (DFS) after concurrent chemotherapy with BPs.

## Case report

**Fig. 1:**
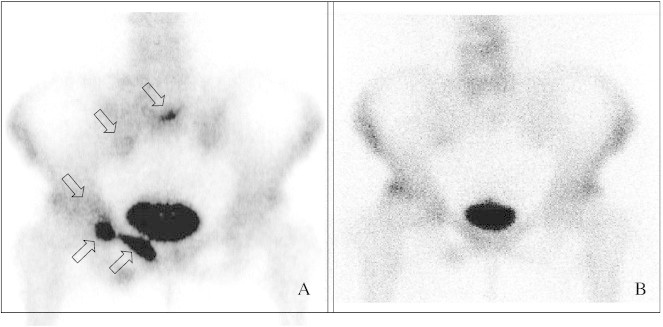
Technetium-99m skeletal scintigraphy of (A) multiple bone metastases 4 months after primary treatment and (B) 41 months after the last administration of pamidronate. Arrows indicate the bone metastases.

**Fig. 2:**
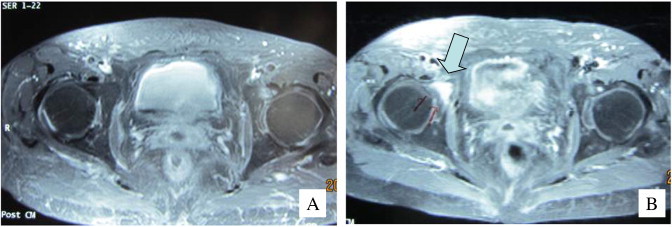
T1-weighted magnetic resonance imaging with contrast enhancement (A) at the time of completion of the primary treatment and (B) 4 months after primary treatment. Arrows indicate 1 of the new lesions.

A 66-year-old female patient first presented to our hospital in 2005 with lower abdominal pain and macroscopic hematuria. The patient was diagnosed with cervical cancer (FIGO stage IVa), and histological analysis determined squamous cell carcinoma. Routine computed tomography (CT) did not detect any apparent lymph node or distant metastases. Primary concurrent chemoradiotherapy (CCRT) consisting of whole-pelvis and paraaortic external beam radiotherapy of 50 and 45 Gy/25 fractions, respectively, was conducted with high-dose-rate intracavitary brachytherapy consisting of 25 Gy/5 fractions. The chemotherapy protocol consisted of intrauterine arterial administration of cisplatin (70 mg/m^2^) every 3 weeks but was only conducted once due to the development of grade 4 neutropenia. Weekly intravenous (IV) administration of cisplatin with radiotherapy had been the standard mode of administration for several years, because there were no randomized trials that supported the superiority of intra-arterial (IA) infusion of cisplatin with radiation. However, with regard to the primary treatment of stage IVa cervical cancer, IA infusion of cisplatin, followed by radical hysterectomy for local control, DFS, and overall survival (OS), was superior to IV administration in the neoadjuvant setting of our series of 3 phase II clinical trials. This infusion technique was also examined at another institution during CCRT; therefore, we adopted the IA infusion of cisplatin with radiotherapy in this case after obtaining written informed consent. Four months after completion of the primary treatment, the patient complained of slight right inguinal pain. Skeletal scintigraphy revealed multiple pelvic bone metastases (Fig. 1A) while magnetic resonance imaging (MRI) revealed a tumor that was not observed at the time of completion of the primary treatment (Fig. 2B). Laboratory tests showed elevated serum alkaline phosphatase (ALP) levels of 357 mIU/ml and N-telopeptide (NTx) levels of 26.7 nM BCE/L. As the pelvic metastatic lesions were located in the previous radiation field, concurrent chemotherapy with BP was conducted. The chemotherapy regimen consisted of intravenous nedaplatin, a cisplatin analogue, at a dose of 80 mg/m^2^ every month for 6 cycles. Because zoledronic acid (ZOL) was not available in Japan at the time, weekly intravenous treatment with pamidronate (15 mg) was administered for 12 months beginning on the first day of nedaplatin treatment. The right inguinal pain was relieved 2 months after the initiation of the treatment. Follow-up skeletal scintigraphy showed no abnormal lesions, and the serum levels of ALP and NTx declined to the normal range 41 months after the last administration of pamidronate (Fig. 1B). The patient is currently alive without any recurrence 89 months after the first diagnosis of bone metastases.

## Discussion

Weekly IV administration of cisplatin with radiotherapy has been the standard mode of administration. However, in the SWOG 8797 trial (Peters et al., 2000), concurrent chemoradiotherapy consisting of a bolus dose of cisplatin (70 mg/m^2^) and a 96-hour infusion of fluorouracil (1000 mg/(m^2^ · d)) every 3 weeks for 4 cycles improved progression-free survival and OS in the adjuvant setting after surgery. In the neoadjuvant chemotherapy setting of our series of 3 phase II clinical trials (Adachi et al., 2001; Tsubamoto et al., 2012, 2013), IA administration of cisplatin was superior to IV administration for local control, DFS, and OS. Kawase et al. reported concurrent chemoradiotherapy consisting of IA infusion of cisplatin (70 mg/m^2^) and a 96-hour IV infusion of fluorouracil (700 mg/(m^2^ · d)) every 3 weeks for 2 cycles (Kawase et al., 2006). Therefore, we adopted the IA infusion of cisplatin concurrent with radiotherapy in this case after obtaining written informed consent.

The prognosis of patients with primary cervical cancer is poor upon the diagnosis of bone metastases.Blythe et al. (1975) reported that 96% of 55 cases had died within 18 months of diagnosis, while Matsuyama et al. (1989) reported that 60% of 48 cases had died within 6 months. Only 3 patients, who had received radiotherapy followed by chemotherapy, survived over 2 years with the longest survivor having lived for 3 years. Thanapprapasr et al. (2010) reported that the median survival time for patients with cervical cancer and bone metastases was 7 months, and among 51 patients, only 1 patient with pelvic-only metastases lived for more than 5 years, having died 90 months after the diagnosis of bone metastases, following an undescribed treatment schedule.

The aim of treatment for bone metastases is usually palliation. The major clinical presentation is pain, and, to achieve pain relief, radiation therapy or administration of BPs, denosumab, or strontium-89 is usually conducted. More than half of the cervical cancer cases show extraskeletal distant metastases (Thanapprapasr et al., 2010), and systemic treatment of chemotherapy is also conducted. Despite integrated treatment and care for bone metastases, patient survival is short and hospice enrollment is discussed.

In the present case, the metastatic lesions were present within a previously irradiated zone of primary external radiation, and additional radiation therapy was not applicable. Early occurrence of the bone metastases after CCRT was associated with a poor response to the chemotherapy regimen and a poor prognosis (Monk et al., 2005; Tanioka et al., 2011).

The anti-cancer activity of BPs in a randomized clinical trial (RCT) was first demonstrated by Diel et al. in 1998. The incidence of distant bone metastases following breast cancer was reduced by a daily oral administration of clodronate, a first-generation BP, for 2 years. Since this report, numerous RCTs have been conducted using BPs in cases of breast cancer and other solid tumors (Gnant and Clézardin, 2012). In the Medical Research Council PR05 and PR04 randomized controlled trials, use of clodronate improved OS in metastatic prostate cancer patients (Dearnaley et al., 2009). Recent reports of ABCSG-12, ZO-FAST, and AZURE trials suggest synergic anti-cancer effects of ZOL and standard treatments in postmenopausal women with breast cancer, although the precise mechanisms behind this effect remains unknown (Gnant et al., 2009; Coleman et al., 2011, 2013). The anti-cancer benefits of ZOL were also demonstrated in metastatic bladder and lung cancer (Zaghloul et al., 2010; Zarogoulidis et al., 2009).

Preclinical studies have demonstrated the antitumor activity of BPs in the bone marrow microenvironment. By inhibiting the activity of farnesyl diphosphate synthase, a key enzyme in the mevalonate pathway, tumor cells show reduced potential for migration, proliferation, and angiogenesis and increased apoptosis. Decreased osteoclast formation and activity reduces the level of growth factors in the bone marrow microenvironment. As a result, apoptosis of cancer cells is expected with synergic antitumor activity of the cytotoxic drugs. Recent clinical studies showed that the circulating and disseminated tumor cells in the blood and bone marrow, respectively, which are relatively dormant and escape cytotoxic drug activity, were reduced by the administration of BPs (Gnant and Clézardin, 2012).

Recently, we surveyed metastatic lesions after serological or radiological recurrence by routine use of 18F-fluorodeoxyglucose positron emission tomography–CT (PET/CT). Because PET is more sensitive than CT and more specific than MRI, early detection of bone metastases was increased at our institute.

Though irradiated bone-only metastases in primary cervical cancer are extremely rare, the combination of chemotherapy with BP treatment might be useful for controlling bone metastases in cervical cancer.

## Conflict of interest statement

The authors have no financial conflicts of interest to disclose.

## References

[bb0025] Abdul-Karim F.W., Kida M., Wentz W.B., Carter J.R., Sorensen K., Macfee M. (1990). Bone metastasis from gynecologic carcinomas: a clinicopathologic study. Gynecol. Oncol..

[bb0050] Adachi S., Ogasawara T., Wakimoto E., Tsuji Y., Takemura T., Koyama K. (2001). Phase I/II study of intravenous nedaplatin and intraarterial cisplatin with transcatheter arterial embolization for patients with locally advanced uterine cervical carcinoma. Cancer.

[bb0020] Blythe J.G., Cohen M.H., Buchsbaum H.J., Latourette H.B. (1975). Bony metastases from carcinoma of cervix. Occurrence, diagnosis, and treatment. Cancer.

[bb0105] Coleman R.E., Marshall H., Cameron D., Dodwell D., Burkinshaw R., Keane M. (2011). Breast-cancer adjuvant therapy with zoledronic acid. N. Engl. J. Med..

[bb0100] Coleman R., de Boer R., Eidtmann H., Llombart A., Davidson N., Neven P. (2013). Zoledronic acid (zoledronate) for postmenopausal women with early breast cancer receiving adjuvant letrozole (ZO-FAST study): final 60-month results. Ann. Oncol..

[bb0090] Dearnaley D.P., Mason M.D., Parmar M.K., Sanders K., Sydes M.R. (2009). Adjuvant therapy with oral sodium clodronate in locally advanced and metastatic prostate cancer: long-term overall survival results from the MRC PR04 and PR05 randomised controlled trials. Lancet Oncol..

[bb0085] Diel I.J., Solomayer E.F., Costa S.D., Gollan C., Goerner R., Wallwiener D. (1998). Reduction in new metastases in breast cancer with adjuvant clodronate treatment. N. Engl. J. Med..

[bb0030] Disibio G., French S.W. (2008). Metastatic patterns of cancers: results from a large autopsy study. Arch. Pathol. Lab. Med..

[bb0040] Gnant M., Clézardin P. (2012). Direct and indirect anticancer activity of bisphosphonates: a brief review of published literature. Cancer Treat. Rev..

[bb0095] Gnant M., Mlineritsch B., Schippinger W., Luschin-Ebengreuth G., Pöstlberger S., Menzel C. (2009). Endocrine therapy plus zoledronic acid in premenopausal breast cancer. N. Engl. J. Med..

[bb0010] Hage W.D., Aboulafia A.J., Aboulafia D.M. (2000). Incidence, location, and diagnostic evaluation of metastatic bone disease. Orthop. Clin. North Am..

[bb0005] Hong J.H., Tsai C.S., Lai C.H., Chang T.C., Wang C.C., Chou H.H. (2004). Recurrent squamous cell carcinoma of cervix after definitive radiotherapy. Int. J. Radiat. Oncol. Biol. Phys..

[bb0065] Kawase S., Okuda T., Ikeda M., Ishihara S., Itoh Y., Yanagawa S. (2006). Intraarterial cisplatin/nedaplatin and intravenous 5-fluorouracil with concurrent radiation therapy for patients with high-risk uterine cervical cancer. Gynecol. Oncol..

[bb0070] Matsuyama T., Tsukamoto N., Imachi M., Nakano H. (1989). Bone metastasis from cervix cancer. Gynecol. Oncol..

[bb0075] Monk B.J., Huang H.Q., Cella D., Long H.J., Gynecologic Oncology Group Study (2005). Quality of life outcomes from a randomized phase III trial of cisplatin with or without topotecan in advanced carcinoma of the cervix: a Gynecologic Oncology Group Study. J. Clin. Oncol..

[bb9000] Moriwaki S. (2007). Pathology of bone metastases between fundamental and clinical aspects.

[bb0015] Nobunaga T., Yamasaki M., Tanaka F., Okamoto E., Hisamatsu K., Ohama K. (1988). A clinicopathological study on bone metastases of cervical carcinoma. Nihon Gan Chiryo Gakkai Shi.

[bb0045] Peters W.A., Liu P.Y., Barrett R.J., Stock R.J., Monk B.J., Berek J.S. (2000). Concurrent chemotherapy and pelvic radiation therapy compared with pelvic radiation therapy alone as adjuvant therapy after radical surgery in high-risk early-stage cancer of the cervix. J. Clin. Oncol..

[bb0080] Tanioka M., Katsumata N., Yonemori K., Kouno T., Shimizu C., Tamura K. (2011). Second platinum therapy in patients with uterine cervical cancer previously treated with platinum chemotherapy. Cancer Chemother. Pharmacol..

[bb0035] Thanapprapasr D., Nartthanarung A., Likittanasombut P., Na Ayudhya N.I., Charakorn C., Udomsubpayakul U. (2010). Bone metastasis in cervical cancer patients over a 10-year period. Int. J. Gynecol. Cancer.

[bb0055] Tsubamoto H., Kanazawa R., Inoue K., Ito Y., Komori S., Maeda H. (2012). Fertility-sparing management for bulky cervical cancer using neoadjuvant transuterine arterial chemotherapy followed by vaginal trachelectomy. Int. J. Gynecol. Cancer.

[bb0060] Tsubamoto H., Maeda H., Kanazawa R., Ito Y., Ohama N., Hori M. (2013). Phase II trial on neoadjuvant intravenous and trans-uterine arterial chemotherapy for locally advanced bulky cervical adenocarcinoma. Gynecol. Oncol..

[bb0110] Zaghloul M.S., Boutrus R., El-Hossieny H., Kader Y.A., El-Attar I., Nazmy M. (2010). A prospective, randomized, placebo-controlled trial of zoledronic acid in bony metastatic bladder cancer. Int. J. Clin. Oncol..

[bb0115] Zarogoulidis K., Boutsikou E., Zarogoulidis P., Eleftheriadou E., Kontakiotis T., Lithoxopoulou H. (2009). The impact of zoledronic acid therapy in survival of lung cancer patients with bone metastasis. Int. J. Cancer.

